# Predictive Validity of Psychometrically Assessed Schizotypy for Psychopathology Dimensions and Functioning in an 8-Year Multiwave Study

**DOI:** 10.1093/schbul/sbae140

**Published:** 2025-03-04

**Authors:** Neus Barrantes-Vidal, Thomas R Kwapil

**Affiliations:** Departament de Psicologia Clínica i de la Salut, Universitat Autònoma de Barcelona, Barcelona, Spain; Centro de Investigación Biomédica en Red de Salud Mental, Instituto de Salud Carlos III, Madrid, Spain; Department of Psychology, University of Illinois at Urbana–Champaign, Champaign, IL, USA

**Keywords:** psychosis, schizophrenia, high-risk, psychosis-proneness, negative symptoms, developmental trajectories

## Abstract

**Background and Hypothesis:**

Although the psychometric high-risk method based on schizotypy has proven to be a highly cost-effective strategy for unraveling etiological factors for schizophrenia-spectrum disorders, there is a paucity of longitudinal studies with nonclinical populations. This study analyzed the predictive validity of positive and negative schizotypy in a longitudinal project (Barcelona Longitudinal Investigation of Schizotypy; BLISS) spanning a total of 7.8 years.

**Study Design:**

At Time 1 (T1), 547 college students completed the Wisconsin Schizotypy Scales. We re-assessed subsamples (oversampled for high schizotypy to ensure variability) at 4 re-assessments. This study reports psychopathology, psychological, and functional outcomes assessed with self-report and interview (CAARMS, Negative Symptom Manual, SCID-II Cluster A) measures at T4 (*n* = 89; 4.4 years after T1) and self-report measures at T5 (*n* = 169; 7.8 years after T1). T1 positive and negative schizotypy were entered simultaneously as predictors in linear regression models.

**Study Results:**

Positive schizotypy predicted positive symptoms at T4, whereas negative schizotypy predicted interview-rated negative symptoms and schizoid personality traits (even when controlling for mood and avoidant personality), and impaired social and global functioning. Both dimensions predicted suspiciousness, and schizotypal and paranoid personality traits, as well as low self-esteem and depression. Similarly, both dimensions predicted suspiciousness, depression, and poor social support at T5, whereas only positive schizotypy predicted low self-esteem, anxiety, and perceived stress.

**Conclusions:**

Both schizotypy dimensions consistently showed a meaningful pattern of hypothesized differential and overlapping predictions, which supports their validity as distinct dimensions and their predictive validity in nonclinical samples.

## Introduction

Schizotypy is conceptualized as the phenotypic expression of the developmental vulnerability for schizophrenia that is expressed across a dynamic continuum of personality traits and symptoms ranging from subclinical impairment to full-blown schizophrenia.^1–6^ Schizotypy provides a useful and unifying framework for examining the etiology, development, and expression of schizophrenia-spectrum psychopathology.^7^ Schizotypy has a heterogeneous multidimensional structure, being positive (odd beliefs, perceptual anomalies, and paranoia), negative (anhedonia, avolition, flattened affect, alogia, and anergia), and disorganized (disruptions in the organization and expression of thought, emotion, behavior) dimensions the most readily identified. This is supported by current conceptualizations,^4^ confirmatory factor analysis,^8,9^ psychometric network models,^10,11^ recent scale development,^12,13^ and multicultural studies.^14,15^

The psychometric high-risk method is a cost-effective strategy involving the identification of psychosis-prone individuals based on schizotypy questionnaires drawn from nonclinically ascertained samples. It avoids the confounds associated with clinical status and provides explanatory power to understand variation in normal behavior as well as risk and resilience processes and trajectories.^7,16,17^ Its utility and validity have been demonstrated in cross-sectional studies with nonclinical young adults showing that positive and negative schizotypy are meaningfully associated with distinct as well as overlapping symptoms and impairment.^18–26^

However, there is a paucity of longitudinal studies. The pioneering investigation by Chapman et al^27^ employed the psychometric-high strategy in a sample of college students identified by scores on the Wisconsin Schizotypy Scales (WSS), including the Perceptual Aberration (PerAb^28^), Magical Ideation (MagicId^29^), and Physical Anhedonia (PhyAnh^30^) Scales. They reassessed 95% of their 534 participants at a 10-year follow-up. In the re-analysis of their findings, Kwapil et al^25^ examined the predictive validity of the psychometrically identified positive and negative schizotypy as assessed by the WSS, including the addition of the Revised Social Anhedonia Scale (SocAnh; Eckblad, Chapman, Chapman, Mishlove, unpublished questionnaire, 1982). They reported that positive schizotypy uniquely predicted psychotic disorders, whereas both dimensions predicted schizophrenia-spectrum disorders. Positive schizotypy predicted psychotic-like symptoms, paranoid, and schizotypal personality disorder (PD) traits, as well as major depressive and manic/hypomanic episodes, substance use disorders, and mental health treatment at the 10-year follow-up. Negative schizotypy predicted social impairment, schizoid, schizotypal, and paranoid PD traits 10 years later. These findings are striking given that participants were functioning well enough to enroll in a major university at the start of the study and were only part-way into the window of greatest risk for developing psychosis at the time of the follow-up assessments. Transition rates into schizophrenia-spectrum disorders were comparable to those typically seen in genetic high-risk studies.^25^ Overall, findings supported the predictive validity of WSS and showed that schizotypy is a useful phenotype for detecting developmental risk for schizophrenia and related disorders.

Lenzenweger^31^ conducted a 17-year reassessment comparing high PerAb and control participants. The PerAb group exceeded controls on rates of nonaffective psychotic disorders and psychotic symptoms at follow-up. Similarly, Gooding et al^32^ found that high scorers on PerAb/MagicId and on SocAnh displayed more positive symptoms compared with controls 5 years later. Unlike Chapman et al.,^27^ baseline ratings did not predict schizophrenia-spectrum disorders at the follow-up, which was attributed to the fact that their participants had better baseline functioning and that the Chapman’s 10-year follow-up covered a greater period of risk. More recently, Bolinskey et al^33^ conducted a 2-year longitudinal study of high schizotypy scorers on either the PerAb, MagicId, or SocAnh Scales, and a matched control group. They reported that the high schizotypy group met more avoidant, paranoid, schizoid, and schizotypal PD traits and were more likely to meet criteria for these PDs than the comparison group at both the baseline and 2-year reassessment. However, no further studies have examined the predictive validity of schizotypy in nonclinical participants. Note that Barrantes-Vidal et al^34^ provide a summary of findings from psychosis-proneness studies employing multiple high-risk methods.

### BLISS Study

We previously reported findings of the Barcelona Longitudinal Investigation of Schizotypy (BLISS), a prospective project examining risk and protective factors for psychosis in a college student sample initially identified with the WSS scales. Barrantes-Vidal et al^18^ found that Time 1 (T1) positive schizotypy was uniquely associated with Time 2 (T2; 1,7 years after T1) interview ratings of positive symptoms, paranoid, and borderline PD traits, as well as negative affect and negative schemas of self and others. In contrast, negative schizotypy was associated with negative and schizoid symptoms, and with diminished positive schemas of self and others. Moreover, both schizotypy dimensions were associated with schizotypal and avoidant PDs traits, suspiciousness, and impaired functioning. In a 3-year follow-up report (Time 3^35^), positive schizotypy predicted interview ratings of positive symptoms and general psychopathology, as well as self-reported depression and low self-esteem, whereas negative schizotypy predicted interview ratings of schizoid traits, emotional disturbance, and mental health treatment during the past year. Both dimensions predicted schizotypal, paranoid, and avoidant traits, suspiciousness, and impaired functioning.

The primary goal of the present study was to extend previous BLISS reports by examining the extent to which psychometrically identified positive and negative schizotypy assessed at T1 differentially predicted symptoms and impairment at two further longitudinal reassessments (T4 at 4.4 and T5 at 7.8 years after baseline). This study expands on the limited prospective research in this area by: (1) employing dimensional measures of positive and negative schizotypy as predictors, (2) including the interview-based assessment of prodromal symptoms and personality disorders at the 4.4-year reassessment (Time 4; T4), and (3) assessing negative symptoms with multiple interviews at the 4.4-year reassessment, which is important when considering concerns that some interview measures of negative symptoms are saturated with depression and neuroticism—characteristics that are not part of current conceptualizations of negative symptoms.^9,19,35^ Given concerns about the feasibility of capturing negative-like symptoms in nonclinical populations, the ability of negative schizotypy to predict these symptoms was examined controlling for emotional dysregulation. Furthermore, this report discusses the consistency of results across the 4 BLISS prospective re-assessments from baseline spanning a total of 7.8 years.

Consistent with our previous findings, it was hypothesized that positive and negative schizotypy dimensions would be associated with overlapping and differential patterns of symptoms and impairment. Specifically, it was expected that both schizotypy dimensions would predict schizotypal, paranoid, and avoidant PD traits as well as suspiciousness and impaired functioning at T4. It was predicted that positive schizotypy would be specifically associated with positive symptoms, depression, anxiety, and low self-esteem, whereas negative schizotypy was expected to be specifically associated with negative symptoms and schizoid traits. Likewise, we hypothesized that positive schizotypy would predict questionnaire measures of positive schizotypy, suspiciousness, social impairment, depression, stress, and diminished self-esteem, and that negative schizotypy would predict negative schizotypy and social impairment at T5.

## Method

### Participants and Procedure

This study is part of BLISS, a longitudinal project examining schizotypy and risk for psychosis-spectrum psychopathology within a college sample. Supplementary Figure S1) provides a flow diagram describing the selection of study participants. At T1, 589 young adults from psychology courses at Universitat Autònoma de Barcelona completed the WSS, the suspiciousness subscale of the Schizotypal Personality Questionnaire (SPQ^36^), and the positive symptom subscale of the Community Assessment of Psychic Experiences (CAPE^37^). Of these, 42 participants were excluded from the final study due to invalid protocols, leaving 547 participants with usable data (mean age = 20.6; SD = 4.1; 86% female). We invited all 189 participants who had standard scores based upon sample norms of at least 1.0 on the positive or negative schizotypy factors derived from the 3 measures, and 150 randomly selected participants who had standard scores < 1.0 to participate at T2. This provided a continuous distribution of scores on the measures with oversampling of participants with elevated schizotypy ratings. Participants were assigned positive and negative schizotypy factor scores based upon norms from 6137 American young adults.^9^ We conducted in-depth assessments at T2 with 214 participants. Of these, due to funding limitations, we opted to select a smaller sub-sample that retained a similar distribution of schizotypy scores for assessment at T3 (*n* = 103). A more detailed description of selection procedures at T1–T2–T3 is described in previous BLISS reports.^18,19,35^

The present study focuses on T4 and T5. A total of 89 participants (mean age = 24.8 years; SD = 2.7; 62% female) completed the reassessment at T4. The sample included 66 of 75 (88%) participants with elevated schizotypy scores (standardized scores of at least 1.0 on any of the T1 schizotypy measures) and 23 of 28 (82%) with standard scores below 1.0. The mean interval between T1 and T4 assessments was 4.4 years (SD = .3, range 4.0–5.2). T5 did not include highly time-consuming interviews, which made it possible to target all participants assessed at T2 (*n* = 214). We were able to successfully reassess 169 (mean age = 28.0; SD = 2.4; 80.5% female) of T2 participants (78.5%). The mean interval between T1 and T5 was 7.8 years (SD = 0.5 years, range 7.0–9.2), and between T4 and T5 was 3.2 years (SD = .4, range 2.5–4.6). The T5 sample included 113 of 123 (91.9%) participants with elevated schizotypy scores (standardized scores of at least 1.0 on any of the T1 schizotypy measures) and 56 of 91 (61.5%) with standard scores below 1.0 from T2.

### Materials

#### Time 1 Questionnaires

At T1, participants completed the WSS and CAPE^37^ scales, and the SPQ-Suspiciousness^36^ subscale. The WSS is one of the most extensively used instruments for assessing schizotypy. It comprises the Perceptual Aberration (PerAb^28^), Magical Ideation (MagicId^29^), Physical Anhedonia (PhyAnh^30^), and Revised Social Anhedonia Scales (Eckblad, Chapman, Chapman, and Mishlove, unpublished questionnaire, 1982). Two factors (positive and negative schizotypy) have been consistently found to underlie the four scales and account for approximately 80% of their variance in American and Spanish samples.^9,38^ These factor scores were used as predictors of symptoms and impairment at the follow-up assessments. The WSS scales were administered intermixed with an infrequency scale (Chapman LJ, Chapman JP, unpublished questionnaire, 1983) to identify invalid responders.

#### Time 4 Measures

##### Questionnaire Measures

Participants completed the short forms of the WSS (WSS-SF^39^) from which positive and negative schizotypy factor scores were derived following the same procedure as in Kwapil et al^25^ WSS-SF comprise a short version of the 4 original WSS (Perceptual Aberration, Magical Ideation, Physical Anhedonia and Revised Social Anhedonia) and have shown good reliability in college samples (eg, Kwapil et al^38^). Suspiciousness was assessed with the SPQ-Suspiciousness subscale.^36^ Self-esteem was measured with the Rosenberg Self-Esteem Scale.^40^ Anxiety symptoms were assessed with the Beck Anxiety Inventory (BAI^41^), and depressive symptoms with the Beck Depression Inventory-II (BDI-II^42^).

##### Interview measures

Symptoms of clinical risk for psychosis were measured with the Comprehensive Assessment of At-Risk Mental States (CAARMS^43^). The CAARMS is a structured interview that can be used to assess positive symptoms in nonclinical populations.^44^ The CAARMS subscales assessing the severity of positive and negative symptoms were administered. Given concerns that the CAARMS negative symptom rating is strongly associated with depressive symptoms and emotional dysregulation, the Negative Symptom Manual (NSM; Kwapil TR, Dickerson LA, unpublished data, 2001) was employed to assess clinical and subclinical negative symptoms at T4. The NSM is a structured clinical interview that assesses 5 classes of symptoms: Anhedonia, Social Withdrawal, Avolition/Anergia, Affective Flattening, and Alogia. Dimensional ratings of schizophrenia-spectrum PDs were obtained with the Structured Clinical Interview for DSM-IV Axis II Disorders.^45^ Participants were interviewed for Paranoid, Schizoid, Schizotypal, and Avoidant Disorder. Functioning was assessed with the Social and Occupational Functioning Assessment Scale^46^ and the Global Assessment of Functioning.^47^

#### Time 5 Measures

Participants completed the short forms of the WSS (WSS-SF^39^) and the SPQ-Suspiciousness subscale.^36^ Social functioning was assessed with the Multidimensional Scale of Perceived Social Support (MSPSS^48^), a 12-item measure designed to assess perceived social support from sources such as family, friends, and significant others. Self-esteem was measured with the Rosenberg Self-Esteem Scale.^39^ Anxiety symptoms were assessed with the anxiety subscale of the Symptom Checklist-90-Revised (SCL-90-R^49^) and depressive symptoms with BDI-II.^41^ Appraisals of stress from the last month were assessed with the Perceived Stress Scale (PSS^50^).

### Data Analysis

We computed linear regressions to examine whether T1 positive and negative schizotypy dimensions predicted symptoms and functioning at T4 and T5. T1 positive and negative schizotypy dimensional scores were entered simultaneously in the regression analyses to examine their unique effects as predictors of the outcome measures at T4. Note that previous assessments by our group have examined the interaction of the positive and negative schizotypy scores as a predictor over-and-above the main effect.^9,19^ However, we opted against including the interaction as we did not have specific hypotheses, we likely lacked adequate power to detect interaction effects,^51^ and previous studies have provided little to no support for such interactions.

For each predictor in the regression models, the standardized regression coefficient (*β*), semi-partial *r*^*2*^, and effect size *f*^*2*^ are reported. According to Cohen,^52^  *f*^*2*^ values above 0.15 are medium effects and above 0.35 are large effect sizes (however, note that designs that employ oversampling can lead to inflated estimates of effect sizes). Given that many of the continuous dependent variables were skewed (especially measures of psychopathology), maximum likelihood estimation and bootstrap procedures (with 2000 samples) were employed.

## Results

### Time 4 Re-assessment

The means for the 89 participants reassessed at T4 were 0.63 for T1 positive schizotypy (SD = 1.35, range =−1.16 to 3.84) and 0.36 for T1 negative schizotypy (SD = 1.32, range =−1.63 to 5.18). Both factor scores were unimodal and positively skewed. They were not significantly correlated (*r* = .05). Supplementary Table S1 provides descriptive data for the interview and questionnaire measures assessed at T4 and T5, and Supplementary Table S2 reports the bivariate correlations of T4 measures.

Results of linear regressions analyzing the prediction of T4 psychosis-spectrum measures, at-risk symptoms, functioning, self, and mood by T1 positive and negative schizotypy factor scores are reported in Table 1. As previously indicated, T1 positive and negative schizotypy factor scores were entered simultaneously to ascertain their unique contribution as predictors of outcome measures. As hypothesized, both schizotypy factor scores assessed at T1 predicted schizotypal and paranoid PD traits, as well as suspiciousness at T4. In addition, both schizotypy factor scores predicted T4 positive symptoms, although the magnitude for positive schizotypy was greater than that of negative schizotypy. Depression ratings and low self-esteem were also predicted by both dimensions. Anxiety ratings were not predicted by either schizotypy factor score. As expected, negative schizotypy predicted schizoid traits (with a large effect size) and negative symptoms, as assessed by both the CAARMS and NSM, as well as functional impairment, at T4.

**Table 1.** Prediction of T4 Psychosis-Spectrum Symptoms and Personality, Functioning, Self-esteem, and Mood by T1 Positive and Negative Schizotypy Dimensions (*n* = 89)

	T1 Positive Schizotypy			T1 Negative Schizotypy		
Criterion T4	β ^a^	Δ*R*^*2*^	*f* ^2^	β ^a^	Δ*R*^*2*^	*f* ^2^
Psychosis spectrum						
Positive schizotypy	0.366^***^	0.134	**0.16** ^b^	0.168	0.028	.03
Negative schizotypy	−0.158	0.025	0.04	0.564^***^	0.317	.***48***
CAARMS positive symptoms	0.431^**^	0.185	0**.25**	0.230^*^	0.053	.07
CAARMS negative symptoms	0.230^*^	0.053	0.06	0.308^**^	0.095	.11
Negative Symptom Manual	0.148	0.022	0.03	0.553^***^	0.305	***0.46***
Schizotypal personality rating	0.291^*^	0.084	0.10	0.325^**^	0.105	.13
Schizoid personality rating	0.040	0.002	0.00	0.544^**^	0.296	***0.42***
Paranoid personality rating	0.231^*^	0.053	0.06	0.234^*^	0.055	0.06
Avoidant personality rating	0.220	0.048	0.05	0.184	0.034	0.04
SPQ suspiciousness	0.279^**^	0.077	0.09	0.288^**^	0.083	0.10
Functioning						
Social and occupational functioning	−0.143	0.020	0.02	−0.291^*^	0.085	0.09
Global assessment of functioning	−0.125	0.016	0.02	−0.341^**^	0.116	0.13
Mood and self-esteem						
Rosenberg self-esteem	−0.309^**^	0.095	0.11	−0.207^*^	0.043	0.05
Beck Depression Inventory	0.268^*^	0.072	0.09	0.277^*^	0.077	0.09
Beck Anxiety Inventory	0.085	0.007	0.01	0.153	0.023	0.02

^*^ *P* < .05, **P < .01, ***P < .001.

^a^Linear regressions were computed to examine the variance accounted for by T1 positive and negative factor scores in predicting outcome measures at T4; both factor scores were entered simultaneously to ascertain their unique contribution as predictors of outcome measures; maximum likelihood estimation and bootstrap procedures (with 2000 samples) were employed.

^b^According to Cohen,^52^ medium effect sizes in bold (*f*^2^ > 0.15), large effect sizes in bold and italics (*f*^2^ > 0.35).

The present findings, coupled with Barrantes-Vidal,^18^ raise concerns that the CAARMS negative symptom ratings tap emotion dysregulation (anxiety, depression, and avoidance)—features which may be phenomenologically similar to negative schizotypy, but are not components of the construct. Therefore, we examined the correlations of the T4 CAARMS and NSM interview ratings of negative symptoms with the T4 schizoid PD dimensional ratings (presumed to be highly associated with negative symptoms), and with the BAI anxiety, BDI depression, and interview avoidant PD ratings. As seen in Table 2, the NSM had a large bivariate association with schizoid ratings, moderate associations with depression and avoidant ratings, and was unassociated with anxiety. Consistent with our concerns, the CAARMS was surprisingly unassociated with the schizoid dimensional ratings, and moderately to strongly associated with the anxiety, depression, and avoidance measures. We further examined this by computing separate linear regression analyses predicting negative symptoms as measured by the T4 CAARMS and NSM partialling out the variance of emotional dysregulation (ie, avoidant PD, anxiety, and depression symptoms) (Table 3). Results showed that the prediction of T4 CAARMS negative symptoms by T1 negative schizotypy was no longer significant, whereas the prediction of T4 NSM negative symptoms by T1 negative schizotypy remained significant.

**Table 2.** Bivariate Correlations of CAARMS Negative and Negative Symptom Manual (NSM) with Schizoid Personality Disorder Dimensional Scores and Emotional Dysregulation

	T4	
	CAARMS Negative	NSM
**T4 measures**	*R*	*R*
Schizoid PD	.16	***.68*** ^*** a^
Emotional dysregulation		
Anxiety	.34^**^	.09
Depression	***.50*** ^**^	.35^**^
Avoidant PD	.35^**^	.32^**^

*Note*: PD refers to Personality Disorder; Anxiety refers to the Beck Anxiety Inventory (BAI^40^); Depression refers to the Beck Depression Inventory-II (BDI-II^41^); CAARMS Negative refers to the Comprehensive Assessment of At-Risk Mental States (CAARMS^42^) negative symptoms dimension; NSM refers to the Negative Symptom Manual (NSM; Kwapil TR, Dickerson LA, unpublished data, 2001).

^a^Large effect size in bold and italics.

^*^ *P* < .05, **P < .01, ***P < .001.

**Table 3.** Linear Regressions of Time 4 CAARMS Negative Symptoms and Negative Symptom Manual (NSM) Scores Controlling for Emotional Dysregulation (*n* = 89)

	Criterion T4					
Predictors	CAARMS Negative			NSM		
T1 Measures	*β* ^a^	Δ*R*^2^	*f* ^2^	*β* ^a^	Δ*R*^2^	*f* ^2^
Schizotypy dimensions						
Positive schizotypy	.102	.009	.01	.071	.004	.01
Negative schizotypy	.174	.027	.04	.486^***^	.213	***.35*** ^b^
T4 measures						
Emotional dysregulation						
Avoidant personality ratings	.186	.030	.05	.159	.021	.03
Beck depression inventory	.276^*^	.046	.07	.186	.021	.03
Beck anxiety inventory	.154	.017	.03	-.095	.007	.01

^a^Linear regressions were computed to examine the variance accounted for by positive and negative schizotypy factor scores (T1) and emotional dysregulation symptoms (T4) in predicting CAARMS negative and NSM measures at T4; both positive and negative schizotypy factor scores were entered simultaneously to ascertain their unique contribution as predictors of outcome measures; maximum likelihood estimation and bootstrap procedures (with 2000 samples) were employed.

^b^According to Cohen,^52^ medium effect sizes in bold (*f*^2^ >.15), large effect sizes in bold and italics (*f*^2^ >.35).

^*^ *P* < .05, **P < .01, ***P < .001.

Results of the linear regression model examining the prediction of negative symptoms as assessed by the NSM and CAARMS negative subscales are reported in Table 4. T1 negative schizotypy uniquely predicted T4 NSM subscales of anhedonia, avolition/anergia, alogia symptoms, social withdrawal, and affective flattening. In contrast, T1 negative schizotypy only predicted T4 CAARMS negative symptom subscale of anhedonia, and both schizotypy dimensions predicted avolition/anergia symptom subscale.

**Table 4.** Linear Regressions of Time 4 NSM and CAARMS Negative Symptom Subscales (*n* = 89)

	T1 Positive Schizotypy			T1 Negative Schizotypy		
Criterion T4	*β* ^a^	Δ*R*^*2*^	*f* ^*2*^	*β* ^a^	Δ*R*^2^	*f* ^*2*^
NSM						
Anhedonia	0.208	0.043	0.05	0.397^**^	0.157	**0.20** ^b^
Avolition/anergia	0.107	0.012	0.01	0.283^*^	0.080	0.09
Alogia	0.065	0.004	0.00	0.208^*^	0.043	0.05
Social withdrawal	0.012	0.000	0.00	0.566^***^	0.320	***0.47***
Affective flattening	0.159	0.025	0.04	0.521^**^	0.271	***0.39***
CAARMS negative						
Anhedonia	0.064	0.004	0.00	0.384^**^	0.147	**0.17**
Avolition/aaanergia	0.262^*^	0.068	0.08	0.236^*^	0.055	0.06
Alogia	0.232	0.054	0.06	0.147	0.022	0.02

^a^Linear regressions were computed to examine the variance accounted for by positive and negative schizotypy (T1) in predicting anhedonia, social withdrawal, avolition/anergia, affective flattening, and alogia as assessed with the NSM at T4; both positive and negative schizotypy factor scores were entered simultaneously to ascertain their unique contribution as predictors of outcome measures; maximum likelihood estimation and bootstrap procedures (with 2000 samples) were employed.

^b^According to Cohen,^52^ medium effect sizes in bold (*f*^2^ > 0.15), large effect sizes in bold and italics (*f*^2^ > 0.35).

^*^ *P* < .05, **P < .01, ***P < .001.

Binary logistic regressions were conducted to examine dichotomous outcomes at T4. Four (4.5%) participants met a diagnosis of psychosis-spectrum personality disorders: 2 Paranoid, 1 Avoidant, and 1 Schizoid PDs. Neither positive (OR = 1.82, 95%CI = 0.53–6.34) nor negative schizotypy (OR = 1.75, 95% CI = 0.75–4.09) predicted these PD at T4. CAARMS attenuated psychosis syndrome criteria were met by 2 participants (2.2%), and this was again not predicted positive (OR = 3.90, 95% CI = 0.70–21.65) or negative schizotypy (OR = 0.99, 95%CI = 0.12–8.21).

Binary logistic regressions were computed to determine whether T1 positive and negative schizotypy predicted self-reports of mental health treatment (ie, psychopharmacological, psychiatric, or psychological treatment) during the past year or at the time of T4 assessments. Twelve participants (13.5%) reported receiving treatment within the past year at T4. T1 negative schizotypy (OR = 1.68, 95% CI = 1.01–2.80, *P* < .05) predicted mental health treatment during the past year, while positive schizotypy did not (OR = 1.67, 95%CI = 0.85–3.29). Moreover, 6 participants (6.7%) reported that they were receiving treatment at the time of T4 assessments. T1 negative schizotypy (OR = 2.20, 95% CI = 1.04–4.67, *P* < .05) predicted mental health treatment at the time of T4 assessments, but positive schizotypy did not (OR = 0.67, 95%CI = .20–2.26).

### Time 5 Re-assessment

The means for the 169 participants reassessed at T5 were 0.33 for T1 positive schizotypy (SD = 1.35, range = − 1.27 to 5.13) and 0.15 for T1 negative schizotypy factor scores (SD = 1.32, range = − 1.63 to 5.18). Both factor scores were unimodal and positively skewed. They were not significantly correlated (*r* = .09). Supplementary Table S1 provides descriptive data for the interview and questionnaire measures assessed at T5 and Supplementary Table S3 of the bivariate correlations of T5 measures.

Results of linear regressions analyzing the prediction of T5 measures by T1 positive and negative schizotypy factor scores are reported in Table 5. Again, both factor scores were entered simultaneously to ascertain their unique contribution as predictors of outcome measures. As expected, positive and negative schizotypy predicted differential patterns of associations at T5. T1 positive schizotypy predicted T5 positive schizotypy, suspiciousness, impaired social functioning, elevated mood and anxiety symptoms, elevated stress, and diminished self-esteem. T1 negative schizotypy predicted T5 negative schizotypy (on the order of a large effect), suspiciousness, and positive schizotypy (these two with small effect sizes). It also was associated with social impairment and depressive and anxious symptoms.

**Table 5.** Prediction of Time 5 Psychosis-Spectrum Symptoms and Personality, Functioning, Self-esteem, and Mood by Time 1 Positive and Negative Schizotypy dimensions (*n *= 169)

	T1 Positive Schizotypy			T1 Negative Schizotypy		
Criterion T5	*β* ^a^	Δ*R*^2^	*f* ^2^	*β* ^a^	Δ*R*^2^	*f* ^2^
Psychosis spectrum						
Positive schizotypy	0.310^***^	0.095	0.11	0.173^*^	0.030	0.03
Negative schizotypy	-0.039	0.002	0.00	0.558^***^	0.308	***0.44*** ^b^
SPQ suspiciousness	0.371^***^	0.137	**0.17**	0.223^**^	0.049	0.06
Functioning						
Social support	-0.186^*^	0.034	0.04	-0.276^***^	0.075	0.09
Mood and self-esteem						
Rosenberg self-esteem	-0.208^**^	0.043	0.04	-0.054	0.00	0.00
Beck Depression Inventory	0.228^**^	0.051	0.06	0.229^**^	0.052	0.06
SCL-90R Anxiety	0.306^***^	0.092	0.11	0.146^*^	0.021	0.02
Perceived subjective stress	0.311^***^	0.096	0.11	0.135	0.018	0.02

^a^Linear regressions were computed to examine the variance accounted for by T1 positive and negative factor scores in predicting outcome measures at T5; both positive and negative schizotypy factor scores were entered simultaneously to ascertain their unique contribution as predictors of outcome measures; maximum likelihood estimation and bootstrap procedures (with 2000 samples) were employed.

^b^According to Cohen,^52^ medium effect sizes in bold (*f*^2^ >.15), large effect sizes in bold and italics (*f*^2^ >.35).

^*^ *P* < .05, **P < .01, ***P < .001.

## Discussion

This study extends previous BLISS reports by examining the construct and predictive validity of positive and negative schizotypy at 4.4 and 7.8 years. This is the first study, to the best of our knowledge, to report the predictive validity of schizotypy dimensions for positive and negative symptoms assessed with CAARMS in a nonclinical sample. Overall, the schizotypy dimensions showed unique and theoretically meaningful patterns of associations with psychosis-spectrum symptoms. Additionally, the results were consistent with previous reassessments,^18,35^ further supporting their predictive validity.

Consistent with our predictions, positive schizotypy predicted interview-rated positive symptoms 4.4 years later, whereas negative schizotypy predicted interview-rated negative symptoms and schizoid personality traits with large effect sizes, as well as impairment in social and global functioning. Notably, the prediction of negative symptoms remained significant (large effect size) when variance of mood symptoms and avoidant personality were partialled from the analyses of NSM, but not CAARMS, negative symptoms ratings. Additionally, only negative schizotypy predicted concurrent and past-year history of mental health treatment. Notably, both schizotypy dimensions predicted suspiciousness, and schizotypal and paranoid traits, as well as low self-esteem and depression. Regarding T5, both schizotypy dimensions predicted suspiciousness, depression, and poor social support 7.8 years later, whereas only positive schizotypy predicted low self-esteem, anxiety and perceived stress. Supplementary Table S4 shows a summary of results (expressed in effect sizes) across all waves of the BLISS study to provide a perspective of the consistency of findings across the full 7.8-year evaluation period.

Negative schizotypy predicted schizoid PD traits, negative symptoms, and impairment in social and global functioning over a 4.4-year period. In contrast, negative schizotypy did not predict avoidant personality ratings, suggesting that the association with schizoid personality is not merely driven by the asocial behavioral component that these 2 PDs share. The finding that negative schizotypy uniquely predicted T4 schizoid PD traits is consistent with our T2 and T3 results,^18,35^ as well as multiple interview studies reported by Hernández et al^53^ Consistent with the specific and stable association between negative schizotypy and schizoid PD across 3 data waves, and mirroring previous assessments, negative schizotypy predicted interview ratings of diminished social functioning at T4—whereas both positive and negative schizotypy predicted *self*-reported low social support both at T4 and T5. The fact that negative schizotypy robustly predicts interview ratings of social functioning problems in college students is especially striking given that these individuals are functioning well enough as to enroll in college courses. These findings are consistent with previous longitudinal studies conducted with individuals at high psychometric^25^ and clinical risk,^54^ as well as with patients with a first episode of psychosis,^55–58^ showing that negative symptoms were specifically associated with social impairment.

This study made an in-depth examination of negative symptoms in nonclinical participants by using 2 interview measures at T4 and analyzing the potential role of emotional dysregulation in the prediction of these symptoms. Negative schizotypy uniquely and strongly predicted NSM negative symptoms, consistent with findings from Kwapil et al,^9^ Kemp et al,^59^ and Hernández et al.^53^ In contrast, and consistent with our previous reports,^18,35^ CAARMS negative symptoms appeared to be saturated by affective dysregulation, as its prediction was no longer significant once variance for avoidant personality, depression, and anxiety was considered in the model. Furthermore, CAARMS negative symptoms was strong correlated with depression. Some measures of negative schizotypy and symptoms are saturated by neuroticism, depression, and social anxiety. On the surface, these constructs may appear overlapping with negative schizotypy (as they share features such as flattened affect, social disinterest, and anhedonia). In negative schizotypy, diminished positive affect, motivation, and cognition tend to be trait-like and not linked to elevated negative affect, whereas these features are episodic and strongly associated with negative affect in depression. Furthermore, neuroticism, which is characterized by unstable affect, stands in contrast to the diminution of affect, motivation, and social interest characterizing negative schizotypy. Barrantes-Vidal and Kwapil^1^ described that affective dysregulation and negative affect are not part of current conceptualizations of negative schizotypy.

Negative schizotypy was found to be cross-sectionally associated with NSM interview ratings of negative symptoms in larger nonclinical samples of Spanish and American students.^9,59–61^ The present results extend these findings by demonstrating that negative schizotypy prospectively predicts all 5 negative symptoms assessed with the NSM interview 4.4 years later. Specifically, negative, but not positive schizotypy, strongly predicted social withdrawal and affective flattening, moderately predicted anhedonia, and modestly alogia and avolition—as well as CAARMS anhedonia. In contrast, both schizotypy dimensions modestly predicted CAARMS avolition. As reported in (Supplementary Table S5), the negative symptoms assessed by CAARMS and NSM are not identical. Both include alogia, avolition, and anhedonia, but CAARMS allocates social anhedonia and affective flattening to other subscales (Behavioral Change and Emotional Disturbance, respectively). In contrast, NSM symptoms and Schizoid PD criteria considerably overlap in content, consistent with the strong correlation here reported between schizoid symptoms and NSM. Schizoid PD ratings did not correlate with CAARMS negative symptoms, probably because the former, like NSM, includes social withdrawal and affective flattening symptoms. In addition, the NSM interview emphasizes carefully screening out other factors that could account for negative features, such as depression, anxiety, illness, and environmental factors.

Negative schizotypy predicted CAARMS positive symptoms at T4 (with small effect size, whereas positive schizotypy showed a large effect size), whereas it did not at T2 and T3.^19,35^ Nevertheless, the present findings are consistent with previous interview studies. Kemp et al^59^ reported that both positive schizotypy (large effect) and negative schizotypy (small effect) predicted interview ratings of positive symptoms. Likewise, Kwapil et al^25^ reported comparable findings at the 10-year follow-up assessment. These findings are consistent with suggestions dating back to Bleuler^62^ that negative symptoms are the fundamental dysfunctions in schizophrenia-spectrum psychopathology, whereas positive symptoms are transient and cut across multiple forms of psychopathology.

As expected, both schizotypy dimensions predicted schizotypal PD traits, consistent with the mixed nature of this PD.^63^ Also, both dimensions predicted schizotypal PD traits and suspiciousness, consistent with our previous reports^19,35^ and with numerous other interview studies.^9,53^ Positive schizotypy appears to be prominently associated with the ideational component of paranoid beliefs that the world is a threatening place and others are hostile and malevolent, whereas negative schizotypy may tap paranoid personality disorder traits more because of behavior secondary to disinterest in contact and closeness with the world, as opposed to overt paranoid ideation.^59^

Results of the BLISS study add strong support to the validity of positive and negative schizotypy as distinct dimensions that can be measured in nonclinical participants. Indeed, accumulating evidence indicates that these dimensions are associated with distinct etiology, expression, and outcome,^9,59^ and failure to consider schizotypy’s multidimensional structure results in a lack of conceptual clarity and explanatory power.^1^ For example, interview,^59^ questionnaire,^64^ and laboratory^65^ studies demonstrate that using a total schizotypy score accounts for only half of the variance accounted for by a multidimensional approach. That said, current models support the inclusion of a disorganized schizotypy dimension,^1^ as included in the Multidimensional Schizotypy Scale. Disorganized schizotypy involves dysregulations in the organization and expression of thought, speech, behavior, and emotion.^4^ Historically, it has been less studied than positive and negative dimensions even though disorganized symptoms and experiences have been historically considered hallmark expressions of schizophrenia-spectrum psychopathology. Recent research conducted with the disorganized dimension of the MSS indicates overall that this is a distinct component of multidimensional schizotypy uniquely associated with disorganized symptoms, attentional deficits, and poor outcomes as assessed with interviews (eg, Hernández et al^53^; Kemp et al,^59^ Kwapil at al^66^), questionnaires (eg, Kemp et al^67^; Pfarr et al^68^; Stinson et al^69^), and experience sampling methodology (ESM; eg, Hernández et al^70^; Kemp et al^71,72^; Kwapil et al^73^; Hernández et al, submitted). That said, disorganized schizotypy has suffered from both conceptual and measurement limitations. Thus, future research on the predictive validity of schizotypy should take into consideration the role of disorganized schizotypy and its interactions with positive and negative schizotypy.

The current study is not without limitations. The generalizability of the findings may be limited by the use of a predominantly female sample that is not representative of the general population. Furthermore, although we were able to reassess the majority (86%) of participants who completed T3, T4 sample size was still relatively small and this may reduce the robustness of our results. Nevertheless, our findings are consistent with those reported in the studies conducted with the largest nonclinical samples^25,27,32^ thus adding support for the validity and usefulness of the psychometric high-risk approach to improve our knowledge of etiological factors without the confounds related to clinical disorders.

Overall, findings indicate that schizotypy offers a useful phenotype that is associated with the risk of schizophrenia-spectrum psychopathology and the developmental trajectory of vulnerability for psychosis. The study of schizotypy clarifies our understanding of continuities and discontinuities between subclinical and clinical expressions of schizotypy (ie, schizophrenia-spectrum disorders), which should improve our knowledge of the heterogeneity in terms of developmental pathways toward both nonclinical and clinical outcomes, helping to identify compensating or protective factors and thus informing early preventative strategies.^2,3,34^ Furthermore, the psychometric high-risk method complements the clinical high risk (CHR) approach. For example, findings of recent studies examining schizotypy in CHR individuals demonstrate that schizotypy is a valid screening method that improves the predictive power of CHR criteria.^74–76^ However, as indicated by research by Flüeckiger et al,^75^ a deeper understanding of the association between schizotypy dimensions, basic symptoms and ultra high-risk symptoms is needed to adequately portray the specific combinations of trait- and symptom-dimensions that are significantly predictive of risk for psychotic disorders. Finally, as pointed out by Oezgen and Grant,^77^ it must be noted that differences in the assessment of schizotypy across studies might impact their predictive validity of psychopathological manifestations, as some (eg, Oxford-Liverpool Inventory of Feelings and Experiences^78^) were designed to capture trait features, whereas others (eg, WSS) focused on deviant experiences and the high-risk aspect of schizotypy. That said, there is considerable overlap among the scales in content domains and even individual items.

## Supplementary Material

Supplementary material is available at https://academic.oup.com/schizophreniabulletin/.

sbae140_suppl_Supplementary_Material
