# Supplementary material for: Predictive Validity of Psychometrically Assessed Schizotypy for Psychopathology Dimensions and Functioning in an 8-Year Multiwave Study
Source: Schizophr Bull. 2025 Mar 4;51(Suppl 2):S115–25. doi: 10.1093/schbul/sbae140 (PMC11879525; doi:10.1093/schbul/sbae140)
Supplement: sbae140_suppl_Supplementary_Material [file sbae140_suppl_supplementary_material.docx]

**Supplementary material**

**Figure S1. Flow diagram describing the selection of study participants of the Barcelona Longitudinal Investigation of Schizotypy (BLISS).**


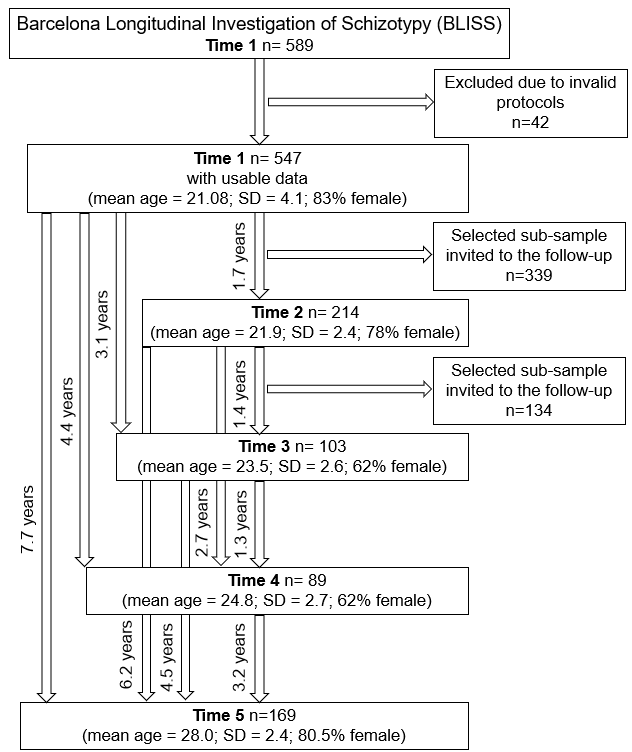


**Table S1.** **Descriptive statistics for quantitative dependent measures of psychosis-spectrum personality and symptoms, mood, self-esteem and impairment at Time 4 and Time 5 re-assessments.**

| \| **Time 4 Measures** \| Mean \| SD \| Range \| Alpha^a^ \| \| --- \| --- \| --- \| --- \| --- \| \| Psychosis Spectrum  SPQ suspiciousness \| 1.25 \| 1.53 \| 0-7 \| .69 \| \| CAARMS positive symptoms \| 1.17 \| 1.96 \| 0-9 \| - \| \| CAARMS negative symptoms \| 1.63 \| 2.46 \| 0-12 \| - \| \| NSM negative symptoms \| 1.92 \| 2.90 \| 0-13 \| - \| \| Schizotypal personality ratings \| 1.08 \| 1.77 \| 0-8 \| - \| \| Schizoid personality ratings \| 1.02 \| 1.94 \| 0-11 \| - \| \| Paranoid personality ratings \| 1.65 \| 2.30 \| 0-12 \| - \| \| Avoidant personality ratings \| 2.01 \| 2.48 \| 0-10 \| - \| \| Functioning \|  \|  \|  \|  \| \| Social and occupational functioning \| 85.8 \| 9.86 \| 50-99 \| - \| \| Global assessment of functioning \| 82.2 \| 12.89 \| 50-100 \| - \| \| Mood and Self-esteem \|  \|  \|  \|  \| \| Rosenberg total \| 22.9 \| 5.16 \| 9-30 \| .88 \| \| Beck depression inventory \| 5.64 \| 6.58 \| 0-33 \| .89 \| \| Beck anxiety inventory \| 5.54 \| 5.78 \| 0-39 \| .88 \| \| **Time 5 Measures** \| Mean \| SD \| Range \| Alpha^a^ \| \| Psychosis Spectrum \|  \|  \|  \|  \| \| Positive Schizotypy \| -0.70 \| 0.42 \| -1.17-2.44 \| - \| \| Negative Schizotypy \| -0.08 \| 0.96 \| -1.06-5.02 \| - \| \| SPQ suspiciousness \| 1.44 \| 1.57 \| 0-8 \| .70 \| \| Functioning \|  \|  \|  \|  \| \| Social support \| 73.26 \| 11.24 \| 13-84 \| .92 \| \| Mood and Self-esteem \|  \|  \|  \|  \| \| Rosenberg self-esteem \| 22.41 \| 5.10 \| 2-30 \| .88 \| \| Beck Depression Inventory \| 5.47 \| 6.16 \| 0-35 \| .89 \| \| Anxiety Symptom Checklist Revised \| 5.40 \| 4.75 \| 0-28 \| .83 \| \| Perceived Stress \| 20.23 \| 7.57 \| 5-43 \| .86 \| |  |  |  |  |
| --- | --- | --- | --- | --- | --- | --- | --- | --- | --- | --- | --- | --- | --- | --- | --- | --- | --- | --- | --- | --- | --- | --- | --- | --- | --- | --- | --- | --- | --- | --- | --- | --- | --- | --- | --- | --- | --- | --- | --- | --- | --- | --- | --- | --- | --- | --- | --- | --- | --- | --- | --- | --- | --- | --- | --- | --- | --- | --- | --- | --- | --- | --- | --- | --- | --- | --- | --- | --- | --- | --- | --- | --- | --- | --- | --- | --- | --- | --- | --- | --- | --- | --- | --- | --- | --- | --- | --- | --- | --- | --- | --- | --- | --- | --- | --- | --- | --- | --- | --- | --- | --- | --- | --- | --- | --- | --- | --- | --- | --- | --- | --- | --- | --- | --- | --- | --- | --- | --- | --- | --- | --- | --- | --- | --- | --- | --- | --- | --- | --- | --- | --- | --- | --- | --- | --- | --- | --- | --- | --- | --- | --- | --- | --- | --- |

^a^ Coefficient alpha reported for questionnaire measures only.

SD = Standard Deviation.

|  | Negative Schizotypy | CAARMS positive symptoms | CAARMS negative symptoms | Negative Symptom Manual | Schizotypal personality rating | Schizoid personality rating | Paranoid personality rating | Avoidant personality rating | SPQ suspiciousness | Social and occupational functioning | Global assessment functioning | Rosenberg self-esteem | Beck Depression Inventory | Beck Anxiety Inventory |
| --- | --- | --- | --- | --- | --- | --- | --- | --- | --- | --- | --- | --- | --- | --- |
| Positive Schizotypy | ,312^**^ | ,436^**^* | 0,143 | ,353^**^ | ,546^***^ | ,348^**^ | ,313^**^ | 0,112 | ,471^***^ | -0,199 | -0,145 | -,210^*^ | ,277^**^ | 0,078 |
| Negative Schizotypy |  | 0,163 | 0,060 | ,406^***^ | ,317^**^ | ,546^***^ | 0,148 | -0,040 | ,235^*^ | -,272^**^ | -,229^*^ | -0,024 | ,218^*^ | 0,079 |
| CAARMS positive symptoms |  |  | ,391^***^ | ,514^***^ | ,573^***^ | ,296^**^ | ,373^***^ | ,358^**^ | ,292^**^ | -,293^**^ | -,293^**^ | -,413^***^ | ,311^**^ | 0,011 |
| CAARMS negative symptoms |  |  |  | ,380^***^ | ,363^***^ | 0,159 | ,353^**^ | ,345^**^ | 0,164 | -0,444*** | -,583^***^ | -,453^***^ | ,494^***^ | ,343^**^ |
| Negative Symptom Manual |  |  |  |  | ,624^***^ | ,664^***^ | ,409^***^ | ,356^**^ | ,401^***^ | -,470^***^ | -,467^***^ | -,387^***^ | ,337^**^ | 0,081 |
| Schizotypal personality rating |  |  |  |  |  | ,498^***^ | ,487^***^ | ,480^***^ | ,397^***^ | -,446^***^ | -,389^***^ | -,478^***^ | ,367^***^ | 0,068 |
| Schizoid personality rating |  |  |  |  |  |  | ,321^**^ | ,211^*^ | ,329^**^ | -,445^***^ | -,392^***^ | -0,157 | ,218^*^ | -0,077 |
| Paranoid personality rating |  |  |  |  |  |  |  | ,459^***^ | ,589^***^ | -,264^*^ | -,338^**^ | -,424^***^ | ,534^***^ | ,263^*^ |
| Avoidant personality rating |  |  |  |  |  |  |  |  | 0,142 | -,330^**^ | -,381^***^ | -,679^***^ | ,327^**^ | 0,071 |
| SPQ suspiciousness |  |  |  |  |  |  |  |  |  | -,351^**^ | -,314^**^ | -,307^**^ | ,577^***^ | ,347^**^ |
| Social and occupational functioning |  |  |  |  |  |  |  |  |  |  | ,861^***^ | ,372^***^ | -,448^***^ | -0,124 |
| Global assessment of functioning |  |  |  |  |  |  |  |  |  |  |  | ,437^***^ | -,538^***^ | -,245^*^ |
| Rosenberg self-esteem |  |  |  |  |  |  |  |  |  |  |  |  | -,536^***^ | -,329^**^ |
| Beck Depression Inventory |  |  |  |  |  |  |  |  |  |  |  |  |  | ,505^***^ |

**Table S2. Bivariate Correlations of Measures Assessed at T4.**

* p < .05, ** p < .01, *** p < .001

**Table S3. Bivariate Correlations of Measures Assessed at T5.**

|  | Negative Schizotypy | SPQ suspiciousness | Social support | Rosenberg self-esteem | Beck Depression Inventory | SCL-90R Anxiety | Perceived Subjective Stress |
| --- | --- | --- | --- | --- | --- | --- | --- |
| Positive Schizotypy | ,326^***^ | ,459^***^ | -,237^**^ | -,228^**^ | ,306^***^ | ,280^**^ | ,344^**^ |
| Negative Schizotypy |  | ,312^***^ | -,433^***^ | -,157^*^ | ,306^***^ | ,085 | ,249^**^ |
| SPQ suspiciousness |  |  | -,323^***^ | -,344^***^ | ,482^***^ | ,457^***^ | ,510^***^ |
| Social support |  |  |  | ,311^***^ | -,334^***^ | -,209^**^ | -,280^***^ |
| Rosenberg self-esteem |  |  |  |  | -,577^***^ | -,324^***^ | -,574^***^ |
| Beck Depression Inventory |  |  |  |  |  | ,528^***^ | ,712^***^ |
| SCL-90R Anxiety |  |  |  |  |  |  | ,618^***^ |

* p < .05, ** p < .01, *** p < .001

**Table S4. Effect Sizes for Longitudinal Associations of Positive and Negative Schizotypy with Symptoms and Functioning Across Four Waves.**

|  | T1 Positive Schizotypy | | | | T1 Negative Schizotypy | | | |
| --- | --- | --- | --- | --- | --- | --- | --- | --- |
|  | Wave # | | | | Wave # | | | |
|  | T2 | T3 | T4 | T5 | T2 | T3 | T4 | T5 |
| Psychosis Spectrum | | | | |  | | | |
| Positive Schizotypy |  | **S** | **M** | **S** |  | S | S | **S** |
| Negative Schizotypy |  | (S) | (S) | (S) |  | **L** | **L** | **L** |
| Suspiciousness | **L** | **S** | **S** | **M** | **S** | **S** | **S** | S |
| Positive symptoms CAARMS | **S** | **S** | **M** |  | S | S | **S** |  |
| Negative symptoms CAARMS | **S** | S | S |  | **S** | S | **S** |  |
| Negative Symptoms Manual |  |  | S |  |  |  | **L** |  |
| Schizoid traits SCID-II | **S** | S | S |  | **L** | **L** | **L** |  |
| Schizotypal traits SCID-II | **S** | **S** | **S** |  | **S** | **S** | **S** |  |
| Paranoid traits SCID-II | **L** | **S** | **S** |  | S | **S** | **S** |  |
| Avoidant traits SCID-II | **S** | **S** | S |  | **S** | **S** | S |  |
| Functioning | | | | |  | | | |
| Social & Occupational functioning | **(S)** | **(S)** | (S) |  | **(S)** | **(S)** | (**S**) |  |
| Global functioning | **(S)** | (S) | (S) |  | **(S)** | **(M)** | **(S)** |  |
| Social support |  |  |  | **(S)** |  |  |  | **(S)** |
| Other phenotypes | | | | |  | | | |
| Depressive interview-based symptoms | **S** | **M** |  |  | S | (S) |  |  |
| Depressive self-report symptoms | **S** | **S** | **S** | **S** | S | S | **S** | **S** |
| Anxiety | **M** |  | S | **S** | S |  | S | **S** |
| Self-esteem | **(M)** | **(M)** | **(S)** | **(S)** | **(S)** | (S) | (**S**) | (S) |
| Perceived stress |  | **M** | **S** | **S** |  | S | **S** | S |

Note. Analyses are based on regression analyses in which schizotypy dimensions were entered as simultaneous predictors. Letters indicate effect sizes based on *f^2^* ^1^: L = large (> .35), M = medium (.15 to .34), S = small (.02 to .14). Values in parentheses indicate inverse associations. Missing values indicate measure not administered. Bold letters indicate reported statistical significance.

**Table S5. Comparison of negative symptoms features and their assessment with the NSM and the CAARMS interview measures with symptoms of Schizoid PD according to the DSM-V.**

|  | **NSM** |  | **CAARMS negative** |  | **Schizoid PD** |
| --- | --- | --- | --- | --- | --- |
| **Negative symptom** |  |  |  |  |  |
| Alogia | - Poverty of content of speech  - Quality of speech (abstract vs. concrete)  - Impoverished thought processes (nonproductive vs. productive – rich responses vs. vague responses with little content)  - Thought blocking (difficulty in completion of responses – loses the thought)  - Spontaneity (difficulty in producing responses to questions that should not require a great deal of thought or in initiating conversation)  - Lengthy pauses in answering questions that do not appear to be because of productive  thought processes.  - Repetitive or perseverative speech  - Poverty of form of speech  - Restricted quantity  - Muteness  - Incoherence or muttered, garbled, mumbled speech  - Inarticulateness |  | Problems trying to form conversations (i.e., hard to find words, thought blocking); responses to questions vague, or convey little information; long time to respond to questions, but when prompted, displays an awareness of the question. |  |  |
| Avolition/Anergia | Assess lack of will, purpose, and volition in the following areas:  (1) goals/planning (lack of goals or difficulty in meeting goals)  (2) impersistence (difficulty in persisting in short- and long-term tasks)  (3) motivation (apathy, reduced sense of will or purpose)  (4) energy level/psychomotor activity (amount of time spent in aimless activity, reduced or  slow physical movements)  (5) grooming/hygiene |  | Assess lack of  (1) energy (mental and physical)  (2) motivation  (3) will power  (4) physical strength  And if this interferes with activities (e.g., school/work and other everyday tasks). |  |  |
| Anhedonia | Assesses the degree to which the participant anticipates, experiences, and pursues pleasurable experiences from the domains of physical experiences, sensory experiences,  hobbies, recreational interests, occupational and/or school interests |  | Assess the degree to which the participant was able to enjoy social activities/work/study as much as usual; if had noticed a decrease in the level of interest in things the participant usually enjoy; if this interfered with the ability to perform activities (e.g., going to school/work/participating in events). |  | Reduced or absent interest for sexual relationships and pleasure activities. |
| Affective Flattening | Assesses the degree to which the participant reports and demonstrates a lack of affective tone and responsivity. Observations cover appearance, tone of voice, interactions with interviewer, facial expressions, and overall affect. |  | *- Subjective emotional disturbance*  *- Observed blunter affect*  *- Observed inappropriate affect*  Symptoms included in the CAARMS Emotional Disturbance index. |  | - Constricted affect  - Flattened affectivity. |
| Social withdrawal | Assesses the degree to which participants experience disinterest in and withdrawal from interpersonal relationships, including relations with family, intimates, friends, and acquaintances. The scale assesses asociality, *not* antisocial tendencies or social anxiety. |  | *- Social isolation*  Symptom included in the CAARMS Behavioral Change index. |  | - Preference for solitary activities - Lack of close friends or confidants. |

NSM = according to the Negative Symptoms Manual (Kwapil TR, Dickerson LA, unpublished data, 2001).

CAARMS negative = according to the Comprehensive Assessment of At-Risk Mental State^2^ negative symptom dimension.

Schizoid PD = symptoms of Schizoid Personality Disorder according to the DSM-V criteria.

^a^ Note: the following Schizoid Personality Disorder symptoms were not included in the table: *Indifference to criticism* identifying Affective Indifference, and *Lack of desire for intimacy* identifying Social Anhedonia.

**References**

1. Cohen AS, Matthews RA. Primary and secondary negative schizotypal traits in a large non-clinical sample. *Pers Individ Dif* 2010;49(5):419-424.
2. Yung AR, Yuen HP, McGorry PD, et al. Mapping the onset of psychosis: the Comprehensive Assessment of At-Risk Mental States. *Aust N Z J Psychiatry* 2005;39(11-12):964-971.
